# Association between Epstein-Barr virus infection and serum positivity rate of anti-nuclear antibodies in Chongqing, China: A cross-sectional observational study

**DOI:** 10.1097/MD.0000000000039233

**Published:** 2024-08-09

**Authors:** Bei-Ning Ding, Yi-Lin Wu, You-Yu Zhang, Yong-Guo Li

**Affiliations:** a Department of Infectious Diseases, The First Affiliated Hospital of Chongqing Medical University, Chongqing 400016, China.

**Keywords:** anti-nuclear antibodies, autoimmune diseases, Epstein-Barr virus, positivity rate

## Abstract

Epstein-Barr virus (EBV) infects over 95% of the global population and is strongly associated with various autoimmune diseases. Anti-nuclear antibodies (ANA) serve as valuable laboratory biomarkers for screening and supporting the diagnosis of various autoimmune diseases. The aim of this study was to assess the prevalence of EBV infection and its association with ANA. This retrospective study employed standard indirect immunofluorescence assay to determine ANA levels, EBV-specific immunofluorescence assay, or plasma EBV-DNA testing. Demographic data including gender and age were collected to observe variations in EBV infection status and ANA positivity rates among different populations. Incorporating 6492 hospitalized patients who underwent ANA antibody spectrum testing, it was observed that serum positivity rates gradually increased with age. The overall serum positivity rate of ANA in females (25.14%) was significantly higher than that in males (13.76%). Among hospitalized patients undergoing EBV-DNA testing, adults aged 21 to 40 years were least affected by EBV, with a positivity rate of 11.96%; however, as age increased, the positivity rate gradually increased. Among the 5225 patients undergoing EBV antibody spectrum testing, ANA-positive patients exhibited significantly higher serum positivity rates for Epstein-Barr nuclear antigen 1 immunoglobulin G, Epstein-Barr virus early antigen immunoglobulin G, Epstein-Barr virus early antigen immunoglobulin A, and Epstein-Barr virus viral capsid antigen immunoglobulin A antibodies compared to ANA-negative patients (*P* < .001; *P* < .001; *P* = .013; *P* < .001). The EBV-DNA positivity rate in ANA-positive patients was also significantly higher than in ANA-negative patients, yielding the same conclusion (*P* = .012). The positivity rates of ANA antibodies in patients with past EBV infection and reactivation were significantly higher than those in uninfected patients (*P* < .001; *P* = .006). The positivity rate of ANA antibodies in reactivated patients was significantly higher than that in primary infected patients and those with past infections (*P* < .001; *P* < .001). Among ANA-positive patients, the positivity rates of EBV antibody spectrum and EBV-DNA were higher compared to ANA-negative patients. The positivity rates of ANA in patients with past EBV infection and reactivation were higher than those in uninfected patients.

**Key points**

- The serum positivity rates of Epstein-Barr nuclear antigen 1 immunoglobulin G, Epstein-Barr virus early antigen immunoglobulin G, Epstein-Barr virus early antigen immunoglobulin A, and Epstein-Barr virus viral capsid antigen immunoglobulin A antibodies in anti-nuclear antibody (ANA)-positive patients were significantly higher than those in ANA-negative patients. Additionally, the EBV-DNA positivity rate in ANA-positive patients was also significantly higher than in ANA-negative patients.
- The positive rate of ANA antibodies in patients with previous infections and reactivation is significantly higher than that in patients without infection, and the positive rate of ANA antibodies in reactivation patients is significantly higher than that in primary infection patients and patients with previous infections.

## 1. Introduction

The Epstein-Barr virus (EBV) is a member of the human herpesvirus family, composed of 8 viruses distributed across 3 subfamilies (α, β, γ).^[1,2]^ The EBV is a lymphotropic herpesvirus, and its infection is highly prevalent worldwide.^[3]^ EBV was first discovered in Burkitt lymphoma in 1964 and later found to be associated with other types of lymphomas, including Hodgkin lymphoma, non-Hodgkin lymphoma in transplant recipients and human immunodeficiency virus–infected individuals, T-cell lymphomas, and natural killer/T-cell lymphomas. EBV is also associated with epithelial cancers, including nasopharyngeal carcinoma and subsets of gastric cancer.^[4]^ In addition to its association with various forms of human cancers, EBV is also associated with several autoimmune diseases , including systemic lupus erythematosus (SLE), Sjögren’s syndrome, rheumatoid arthritis (RA), and multiple sclerosis (MS). Studies have shown a strong correlation between SLE, with SLE patients exhibiting increased EBV antibody titers and viral DNA.^[5]^ Furthermore, in studies of transcription factor binding to the human genome, the EBV EBNA2 protein binds to regulatory regions associated with SLE and other autoimmune diseases, including MS and RA.^[6]^ The presence of EBV viral DNA in the blood of RA patients is also significantly increased, doubling their risk of developing lymphomas.^[7]^ Evidence from multiple epidemiological studies associates EBV infection with MS, indicating MS as a rare complication of EBV infection.^[8,9]^

Autoantibodies are hallmarks of autoimmunity, among which anti-nuclear antibodies (ANA) historically play a central role.^[10,11]^ ANA comprises a group of autoantibodies targeting various intracellular antigens in different cellular compartments, including nuclear components (chromatin, nucleoli, and nucleoplasm), nuclear membrane components, mitotic spindle apparatus, and cytoplasmic solutes.

The EBV has multiple mechanisms to evade host immune defenses and establish persistent infection.^[12]^ As part of the shared evolutionary history between humans and EBV, the virus has evolved various immune evasion mechanisms, including enveloping itself in membranes derived from host cells (envelopment) and the ability to switch between latent and lytic life stages.^[13,14]^ EBV also produces soluble mediators that interfere with the mobilization of the adaptive immune system.^[15]^ In practical clinical settings, the development of autoimmune diseases often follows persistent viral infections. The course of EBV infection is determined by viral load and the individual’s immune system status, which, in turn, depend on factors such as genetic composition, other infection history, and several environmental factors, all of which may affect an individual’s immune competence to varying degrees.

In this context, the purpose of this study is to assess the positivity rates of EBV infection and ANA-related antibodies, providing researchers and clinicians with more baseline information that will aid in the diagnosis and prevention of EBV-related autoimmune diseases.

## 2. Subject and methods

### 2.1. Ethics approval and consent to participate

This retrospective study received approval from the Ethics Committee of the First Affiliated Hospital of Chongqing Medical University (Approval Number: K2023-217) and was conducted in accordance with the standards outlined in the Helsinki Declaration. Informed consent requirements were waived, and patient identity information was removed prior to the analysis of test results.

### 2.2. Patients and samples

This retrospective study included 6492 inpatients (3306 males and 3186 females) from the First Affiliated Hospital of Chongqing Medical University between June 3, 2019 and December 25, 2022, ranging in age from 12 to 96 years. They underwent tests for ANAs profile, EBV antibody profile, and EBV-DNA quantification. We analyzed the test results in conjunction with demographic variables and described the epidemiology related to the ANA profile, EBV antibody profile, and EBV-DNA.

### 2.3. Laboratory testing

#### 2.3.1. ANA and EBV antibody spectrum detection

We used the HISCL-5000 automated immunoassay system from Sysmex, Kobe, Japan, along with Sysmex-provided ANA panel assay kits. This kit detects multiple ANAs, including anti-double-stranded DNA antibodies, anti-SSA antibodies, anti-Ro-52 antibodies, anti-SSB antibodies, anti-Scl-70 antibodies, anti-Sm antibodies, and anti-U1-snRNP antibodies. For other specific antibodies (e.g., anti-mitochondrial antibody M2 type, anti-nucleosome antibody, anti-histone antibody), we used specialized enzyme-linked immunosorbent assay kits from Bio-Rad, Hercules, California.

Detailed steps are as follows:

Sample preparation: Diluted serum samples were processed according to the HISCL-5000 system’s manual.
Detection process: Processed samples were added to microplates pre-coated with specific antigens and incubated at room temperature for 1 hour.
Washing: Unbound components were washed away using a wash buffer provided by the system.
Detection: Enzyme-labeled secondary antibodies were added and incubated at room temperature for 30 minutes. Substrate solution was then added, and the color reaction was read at 450 nm wavelength. Results were calculated based on the standard curve.

#### 2.3.2. EBV-DNA detection

We used the AGS4800 instrument from Anyu Technology to detect EBV-DNA. The detailed steps are as follows:

Sample preparation: DNA was extracted from serum samples using the QIAamp DNA Blood Mini Kit from Qiagen, Hilden, North Rhine-Westphalia, Germany, following the manufacturer’s instructions.
Quantitative polymerase chain reaction (qPCR) reaction system: The reaction system included a total volume of 20 µL, consisting of 10 µL TAKARA qPCR Master Mix, 1 µL forward primer (10 µM), 1 µL reverse primer (10 µM), 0.5 µL probe (10 µM), 2 µL template DNA, and 5.5 µL nuclease-free water.
Thermal cycling conditions: Initial denaturation at 95°C for 5 minutes, followed by 40 cycles of 95°C for 15 seconds and 60°C for 1 minute using Bio-Rad’s S1000TM thermal cycler.
Detection and data analysis: Fluorescence signals were detected using the QIAquant96PCR instrument from Qiagen. Ct values were quantified using the standard curve.

#### 2.3.3. Types of antibodies detected and definition of ANA positivity

Response: In our study, we used the HISCL-5000 automated immunoassay system to detect a range of ANA, including but not limited to the following:

ANA
Anti-U1-snRNP antibody
Anti-Sm antibody
Anti-SSA antibody
Anti-Ro-52 antibody
Anti-SSB antibody
Anti-Scl-70 antibody
Anti-PM-Scl antibody
Anti-Jo-1 antibody
Anti-centromere protein B antibody
Anti-proliferating cell nuclear antigen antibody
Anti-double-stranded DNA antibody
Anti-nucleosome antibody
Anti-histone antibody
Anti-ribosomal P protein antibody
Anti-mitochondrial antibody M2 type

We adhered to widely accepted international standards and guidelines, defining ANA positivity as an ANA titer greater than 1:80. Although we detected multiple specific antibodies, we standardized our study by defining ANA positivity based solely on broad ANA results. We believe this standard provides reliable and valuable reference results.

#### 2.3.4. Classification of ANA patterns

We have classified ANA patterns based on established standards using indirect immunofluorescence on human epithelial type 2 (HEp-2) cells. The classification is as follows:

Homogeneous: Uniform staining of the entire nucleus.
Speckled: Numerous granular spots within the nucleus.
Nucleolar: Prominent staining of nucleoli.
Cytoplasmic: Staining concentrated within the cytoplasm.
Centromere: Discrete spots corresponding to centromeres.
Cytoplasmic speckled: Granular staining within the cytoplasm.
Cytoplasmic fibrillar: Fibrous staining within the cytoplasm.
Mixed: Presence of multiple patterns mentioned above.
Other: Staining patterns not fitting the above categories.

This classification was performed by trained personnel and verified by a second independent observer to ensure accuracy. These details have been added to the Methods section.

### 2.4. Statistical analysis

Categorical variables are presented as n (%) and compared using the chi-square test. Skewed continuous variables are presented as median (interquartile range), and the Kruskal-Wallis rank sum test is used to pairwise compare the ages among different patient groups with diseases. All statistical analyses were performed using IBM SPSS Statistics software, version 27.0 .

## 3. Results

As shown in Table 1, among the 6492 hospitalized patients undergoing ANA antibody spectrum testing, along with the detection of EBV-related antibody spectrum or EBV-DNA, the actual number of patients for each testing parameter in each age group can be calculated by dividing the number of positive patients by the percentage.

**Table 1:** Age and gender-related differences in EBV antibody profiles, ANA positivity, and EBV-DNA positivity among patients.

	EBNA-1-IgG	EBV-VCA-IgM	EBV-EA-IgG	EBV-EA-IgA	EBV-VCA-IgA	ANA	EBV-DNA
0–20	350 (79.91)	68 (15.49)	51 (11.64)	65 (15.01)	103 (23.79)	71 (13.35)	23 (14.65)
21–40	1457 (92.16)	104 (6.57)	158 (9.99)	185 (11.87)	353 (22.64)	257 (13.49)	67 (11.96)
41–60	1856 (94.55)	78 (3.97)	254 (12.94)	206 (10.60)	478 (24.59)	518 (21.19)	120 (15.38)
>61	1130 (91.42)	31 (2.51)	246 (19.90)	178 (14.57)	407 (33.31)	410 (25.45)	165 (25.35)
Male	2462 (92.31)	131 (4.90)	329 (12.34)	349 (13.25)	707 (26.85)	455 (13.76)	200 (18.25)
Female	2331 (91.38)	150 (5.87)	380 (14.90)	285 (11.29)	634 (25.11)	801 (25.14)	177 (16.83)
Total	4793 (91.86)	281 (5.38)	709 (13.59)	634 (12.29)	1341 (26.00)	1256 (19.35)	375 (17.46)

ANA = anti-nuclear antibodies, EBNA-1-IgG = Epstein-Barr nuclear antigen 1 immunoglobulin G, EBV-DNA = Epstein-Barr virus deoxyribonucleic acid, EBV-EA-IgA = Epstein-Barr virus early antigen immunoglobulin A, EBV-EA-IgG = Epstein-Barr virus early antigen immunoglobulin G, EBV-VCA-IgA = Epstein-Barr virus viral capsid antigen immunoglobulin A, EBV-VCA-IgM = Epstein-Barr virus viral capsid antigen immunoglobulin M.

In the 6492 hospitalized patients undergoing ANA antibody spectrum testing, the serum positivity rates increased gradually with age, being 13.35%, 13.49%, 21.19%, and 25.45%, respectively. The total serum positivity rate of ANA in females (25.14%) was significantly higher than that in males (13.76%).

Among patients undergoing EBV antibody spectrum testing, the serum total positivity rates for Epstein-Barr nuclear antigen 1 immunoglobulin G (EBNA-1-IgG), Epstein-Barr virus viral capsid antigen immunoglobulin M (EBV-VCA-IgM), Epstein-Barr virus early antigen immunoglobulin G (EBV-EA-IgG), Epstein-Barr virus early antigen immunoglobulin A (EBV-EA-IgA), Epstein-Barr virus viral capsid antigen immunoglobulin A (EBV-VCA-IgA), and EBV-DNA were 91.86%, 5.38%, 13.59%, 12.29%, 26.00%, and 17.46%, respectively. The serum positivity rates in males were 92.31%, 4.90%, 12.34%, 13.25%, 26.85%, and 18.25%, respectively. The serum positivity rates in females were 91.38%, 5.87%, 14.90%, 11.29%, 25.11%, and 16.83%, respectively.

Among hospitalized patients undergoing EBV-DNA testing, the age group least affected by EBV was adults aged 21 to 40 years, with a positivity rate of 11.96%. With increasing age, the positivity rate gradually increased.

Further observation of antibody titers and fluorescence patterns of ANA antibody spectrum, as shown in Table 2, revealed various fluorescence patterns in 1256 ANA-positive patients, with speckled and homogeneous cytoplasmic patterns being predominant. ANA positivity was mainly observed at titers of 1:100 and 1:320 among patients.

**Table 2:** The relationship between ANA positivity titers and fluorescence patterns among patients.

	ANA titers 1: 100 (N)	ANA titers 1: 320 (N)	ANA titers 1: 1000 (N)
Speckled pattern	251	87	58
Homogeneous pattern	116	43	38
Nucleolar pattern	96	18	3
Cytoplasmic pattern	84	21	3
Centromere pattern	21	26	0
Cytoplasmic speckled pattern	129	20	0
Cytoplasmic filamentous pattern	15	1	0
Other	55	17	1
Mixed pattern	32	34	8
Total	799	267	111

ANA = anti-nuclear antibodies, N = number of cases.

The positivity rates of EBV antibody profiles differ among patients with different ANA antibody statuses, as illustrated in Table 3. Among ANA-positive patients, the serum positivity rates of EBNA-1-IgG, EBV-EA-IgG, EBV-EA-IgA, and EBV-VCA-IgA antibodies were significantly higher compared to ANA-negative patients (a: *P* < .001; b: *P* < .001; c: *P* = .013; d: *P* < .001). Moreover, ANA-positive patients exhibited a significantly higher EBV-DNA positivity rate compared to ANA-negative patients, supporting the same conclusion (*P* = .012).

**Table 3:** The relationship between different ANA antibody statuses and the positivity rates of EBV antibody profiles.

	EBNA-1 IgG [N (%)]	EBV-VCA-IgM [N (%)]	EBV-EA-IgG [N (%)]	EBV-EA-IgA [N (%)]	EBV-VCA-IgA [N (%)]	EBV-DNA [N (%)]
ANA negative	3863 (91.26)	227 (5.36)	469 (11.08)	491 (11.74)	993 (23.75)	274 (16.37)
ANA positive	930 (94.42)	54 (5.48)	240 (24.37)	143 (14.64)	348 (35.62)	101 (21.31)
*P* value	<.001*	.879	<.001†	.013‡	<.001§	.012

*P* < .05 is considered statistically significant.

ANA = anti-nuclear antibodies, EBNA-1-IgG = Epstein-Barr nuclear antigen 1 immunoglobulin G, EBV-DNA = Epstein-Barr virus deoxyribonucleic acid, EBV-EA-IgA = Epstein-Barr virus early antigen immunoglobulin A, EBV-EA-IgG = Epstein-Barr virus early antigen immunoglobulin G, EBV-VCA-IgA = Epstein-Barr virus viral capsid antigen immunoglobulin A, EBV-VCA-IgM = Epstein-Barr virus viral capsid antigen immunoglobulin M.

* The serum positivity rate of EBNA-1-IgG was significantly higher in ANA-positive patients compared to ANA-negative patients (*P* < .001).

† The serum positivity rate of EBV-EA-IgG was significantly higher in ANA-positive patients compared to ANA-negative patients (*P* < .001).

‡ The serum positivity rate of EBV-EA-IgA was significantly higher in ANA-positive patients compared to ANA-negative patients (*P* = .012).

§ The serum positivity rate of EBV-VCA-IgA was significantly higher in ANA-positive patients compared to ANA-negative patients (*P* < .001).

Among the 5225 patients who underwent EBV antibody profiling, 4 distinct EBV infection statuses were categorized based on antibody combination patterns. Among them, 243 patients were classified as uninfected, 129 as primary infection, 3566 as past infection, and 778 as reactivation infection. As shown in Table 4, the positivity rates of ANA antibodies in patients with past infection and reactivation infection were significantly higher than those in uninfected patients (a: *P* < .001; c: *P* = .006). Additionally, the positivity rate of ANA antibodies in reactivation infection patients was significantly higher than that in both primary infection and past infection patients (b: *P* < .001; d: *P* < .001).

**Table 4:** Relationship between different EBV infection status and ANA antibody positive rate.

Antibody spectrum combination patterns (NA-IgG, VCA-IgM, EA-IgG)	Infection status	ANA negative			ANA positive			*P* value
		N (%)	Male	Age (y) [years, M (P25, P75)]	N (%)	Male	Age (y) [years, M (P25, P75)]	
−/−/−	No infection	218 (89.71)	115	44.0 (24.0–61.25)	25 (10.29)	7	49.0 (28.0–56.0)	<.001; .019 *
−/+/+, −/±, −/−/+	Primary infection	109 (84.50)	55	25.0 (19.0–47.5)	20 (15.50)	5	32.5 (21.25–56.0)	<.001; .036 †
±/−	Past infection	3310 (83.02)	1828	45.5 (30.0–57.0)	677 (16.98)	256	52.0 (38.0–63.5)	.006 <.001 ‡
+/+/+, +/±, ±/+	Reactivation	525 (67.48)	283	34.0 (48.0–64.0)	253 (32.52)	84	42.0 (54.0–66.0)	<.001 <.001 §

ANA = anti-nuclear antibodies, EA-IgG = early antigen immunoglobulin G, EBV = Epstein-Barr virus, NA-IgG = nuclear antigen immunoglobulin G, VCA-IgM = viral capsid antigen immunoglobulin M.

* The comparison between uninfected status and reactivation status yielded a statistically significant difference (*P* < .001). In the uninfection status group, the ANA-positive rate of men was significantly lower than that of women (*P* = .019).

† The comparison between primary infection status and reactivation status yielded a statistically significant difference (*P* < .001). In the primary infection status group, the ANA-positive rate of men was significantly lower than that of women (*P* = .036).

‡ The comparison between uninfected status and past infection status yielded a statistically significant difference (*P* = .006). In the past infection status group, the ANA-positive rate of men was significantly lower than that of women (*P* < .001).

§ The comparison between past infection status and reactivation status yielded a statistically significant difference (*P* < .001). In the reactivation status group, the ANA-positive rate of men was significantly lower than that of women (*P* < .001).

Among the 4 groups with different EBV infection status, the ANA-positive rate of males in these 4 groups was significantly lower than that of females (*P* = .019, *P* = .036, *P* < .001, *P* < .001).

## 4. Discussion

Most autoimmune diseases are chronic conditions with unknown etiology and diverse pathological manifestations. Currently, various factors are considered to play crucial roles in the occurrence and development of these diseases, including genetics, environmental factors, immunity, and hormones. Studies have found compelling evidence indicating that EBV serves as an environmental factor in the pathogenesis of several autoimmune diseases, including SLE, Sjögren’s syndrome, RA, and MS.^[4]^

In early life, most individuals undergo EBV infection and seroconversion. For the majority of infected individuals, latent infection does not adversely affect overall health. However, loss of control during the latent phase or reactivation leading to viral replication may result in the onset of EBV-related diseases.^[16^ ANAs collectively refer to a group of autoantibodies that target various cellular components of the self. The presence of high-titer autoantibodies in the blood is one of the characteristics of autoimmune diseases and serves as an important diagnostic criterion for clinical diagnosis of autoimmune disease. Increasing evidence suggests that autoantibodies can precede symptomatic autoimmune disease by several years.^[17]^ ANA testing holds significant clinical relevance in assessing the activity of autoimmune disease, monitoring treatment efficacy, and guiding clinical medication.^[18]^

In this study, ANA positivity was more common in females than males, and its positivity rate increased gradually with age, consistent with current research findings.^[19–21]^ Compared to the healthy control group with negative ANA serum, ANA-positive patients exhibited higher levels of EBNA-1-IgG, EBV-EA-IgG, EBV-EA-IgA, and EBV-VCA-IgA, showing a similar trend in EBV-DNA detection positivity. Furthermore, we found that the reactivation infection status might be associated with the presence of a high ANA positivity rate, which reinforces the role of the virus in the dysregulation of immune response to self-antigens. This finding is consistent with a study conducted in Italy.^[22]^

We observed a significant increase in the positivity rate of EBV antibody profiles in ANA-positive patients, along with the association between EBV infection status and ANA. Since the trend of ANA may be a marker for increased susceptibility to autoimmune diseases, the similar trend between EBV infection and ANA signifies the potential clinical relevance of our broader findings.

Our study has limitations; it is a single-center cross-sectional study, and we could not assess the comprehensive health status of patients. Some potential confounding factors not included in the analysis might influence the results. Despite being based on data from hospitalized patients, we believe that the results of the large sample analysis are reliable.

In conclusion, our study results indicate an association between ANA-positive patients and EBV infection, reinforcing the link between autoantibodies and EBV immune response. However, we look forward to further research with large-sample, multicenter, and prospective studies to investigate potential mechanisms through experimentation, in order to better understand the role of the virus in autoimmune diseases.

## Author contributions

**Conceptualization:** Bei-Ning Ding, Yong-Guo Li.

**Data curation:** Bei-Ning Ding.

**Investigation:** Bei-Ning Ding, You-Yu Zhang.

**Methodology:** Bei-Ning Ding.

**Project administration:** Bei-Ning Ding.

**Software:** Bei-Ning Ding, Yi-Lin Wu.

**Supervision:** Bei-Ning Ding.

**Visualization:** Bei-Ning Ding.

**Writing – original draft:** Bei-Ning Ding.

**Writing – review & editing:** Yong-Guo Li.
